# Protective effects of sulforaphane on di-n-butylphthalate-induced testicular oxidative stress injury in male mice offsprings via activating Nrf2/ARE pathway

**DOI:** 10.18632/oncotarget.19981

**Published:** 2017-08-07

**Authors:** Zhiqiang Qin, Jingyuan Tang, Peng Han, Xuping Jiang, Chengdi Yang, Ran Li, Min Tang, Baixin Shen, Wei Wang, Chao Qin, Wei Zhang

**Affiliations:** ^1^ Department of Urology, The First Affiliated Hospital of Nanjing Medical University, Nanjing 210009, China; ^2^ Department of Urology, Yixing People’s Hospital, Yixing 214200, China; ^3^ Department of Urology, The Second Affiliated Hospital of Nanjing Medical University, Nanjing 210000, China

**Keywords:** sulforaphane, di-n-butylphthalate, oxidative stress injury, testis, Nrf2

## Abstract

Di-N-butylphthalate (DBP) is one of the most common endocrine-disrupting chemicals which can disrupt human endocrine system, especially in the male reproductive system. Here, this study was aimed to determine whether sulforaphane (SFN) could protect against testicular oxidative stress injury induced by DBP in male mice offsprings. Wild-type (Nrf2^+/+^) and Nrf2-deficient (Nrf2^-/-^) timed-pregnant mice were given DBP orally from embryonic day (E)14.5 to E19.5. Subsequently, the oxidative stress markers were evaluated. Besides, Nrf2, NF-κB, I-kB, HO-1 and NQO-1 expression levels in the testis were measured by immunohistochemical staining or western blot analysis. DBP significantly reduced anogenital distance (AGD) and influenced testes growth in male mice offsprings, while SFN ameliorated these phenotypes. After DBP stimulation, the testicular morphology, testicular cell apoptosis index and the oxidative stress markers exhibited statistical differences compared with Control group, while SFN supplementation showed obvious improvements. In addition, administration of SFN could obviously increase the expression level of Nrf2 and its downstream ARE gene battery, such as HO-1, NQO-1 in the testis. Meanwhile, SFN pretreatment did not confer protection against DBP-induced testicular oxidative stress injury in Nrf2 knockout mice. Therefore, the present findings suggested that SFN could effectively protect against DBP-induced testicular oxidative stress injury through Nrf2/ARE signaling pathways in male mice offsprings.

## INTRODUCTION

Endocrine-disrupting chemicals (EDCs), as one of ubiquitous chemical pollutants, have been considered as a potential risk-factors in male reproductive malformation and infertility, including hypospadias, cryptorchidism, and other birth defects [1, 2]. Amongst all, di-N-butylphthalate (DBP) has been suggested as one of the most critical EDCs, which is extensively used for plasticizers [3]. As DBP doesn’t covalently bound to the plastic matrix, it can leach into surrounding environment [4]. During embryonic period, DBP can cause the oxidative stress injury in a variety of organs, such as brain, lung, and liver. Amid all organs, DBP has the highest affinity for testes and induces the oxidative stress injury, causing male reproductive dysfunction [5, 6]. Our previous studies have suggested DBP could induce oxidative stress injury in testicular Leydig cells [7].

As an important member of the cap’n’collar (CNC) family, nuclear factor erythroid-related factor 2 (Nrf2) is a key transcription factor in the cellular defense system, which contains a conserved basic-region leucine zipper (bZIP) structure [8, 9]. Nrf2 can activate the cellular antioxidant response element (ARE) which is located in the promoters of these target genes to induce gene transcription, thus to enhance adaptive responses to various environmental stressors, such as xenobiotics and oxidative stress [10]. Besides, Nrf2-dependent activation of ARE-driven gene promoters can result in the induction of a series of cytoprotective proteins, including cytoprotective phase II detoxifying enzymes as well as downstream target genes, such as hemeoxygenase 1 (HO-1) and NAD(P)H-quinone oxidoreductase 1 (NQO-1) [11, 12]. Previous studies also have demonstrated that loss and/or dysregulation of Nrf2 might cause the oxidative disruption in testis [13].

Sulforaphane (SFN) is an isothiocyanate abundant in cruciferous vegetables and has proved to be antioxidative via Nrf2 for a range of diseases based on previous studies *in vivo* and *in vitro* [14]. As a widely used Nrf2 activator, SFN has been reported to show protective properties in experimental renal reperfusion injury [15], renal fibrosis [16], cardiovascular diseases [17]. However, few studies focused on the protective role of SFN on DBP-induced testicular toxicity in mice offsprings and its exact mechanism is still unclear.

An animal model of DBP-induced testicular oxidative stress injury was used to investigate whether DBP deteriorated the oxidative damage, particularly in the process of male reproductive formation. Moreover, we selected wild-type (Nrf2^+/+^) mice and Nrf2 knockout (Nrf2^−/−^) mice as experimental subjects to determine whether SFN effectively protected against DBP-induced testicular oxidative stress injury via Nrf2/ARE signaling pathways in male mice offsprings. Previous studies found that the reproductive ability of Nrf2^-/-^ males did not differ from those of wild-type males [13, 18, 19]. Therefore, the present study aimed to examine whether SFN could activate Nrf2 and its target genes to reduce testicular oxidative stress induced by DBP in the mice offsprings.

## RESULTS

### Effect of DBP stimulation and SFN supplementation on anogenital distance (AGD) and testes growth

**Figure 1:**
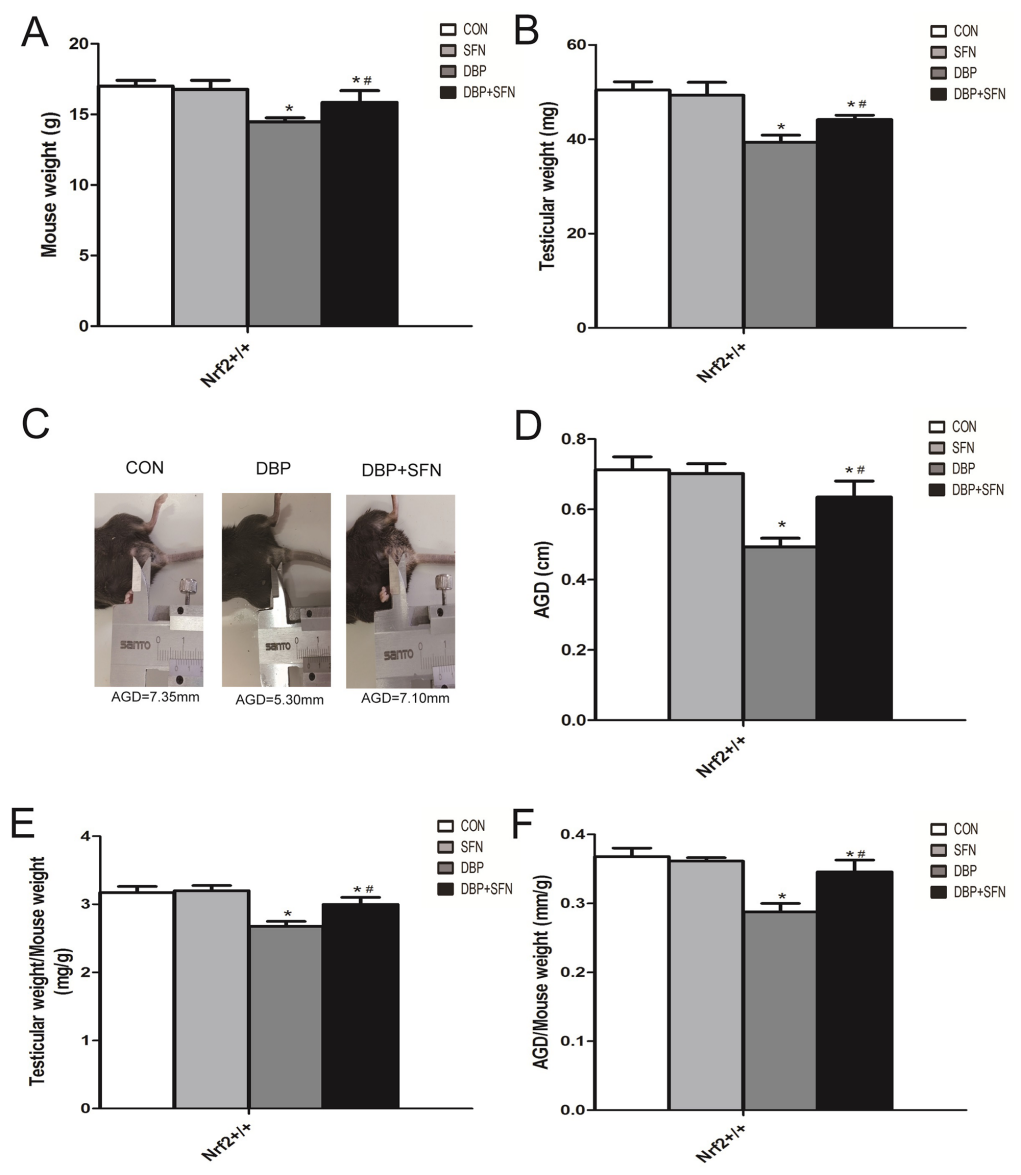
Effect of DBP stimulation and SFN supplementation on AGD and testes growth in Nrf2^+/+^ mice **(A)** Body weight of mouse. **(B)** Testicular weight of mouse. **(C)** Picture examples of AGD. **(D)** AGD of mouse. **(E)** The ratio of testes weight/body weight. **(F)** The ratio of AGD/body weight. Columns represent mean ± SD. *significant difference vs. Control group (P < 0.05); #significant difference vs. DBP treated group (P < 0.05).

As shown in Figure 1, Comparing DBP fed Nrf2^+/+^ mice with Control group, body weight, testes weight and AGD were significantly different. Meanwhile, testis ratio (testes weight/body weight) and AGD index (AGD/body weight) were also reduced in DBP fed Nrf2^+/+^ mice. Interestingly, the supplementation of SFN significantly improved these phenotypes in Nrf2^+/+^ mice.

### SFN preventes DBP-induced testicular oxidative stress injury in testicular morphology

**Figure 2:**
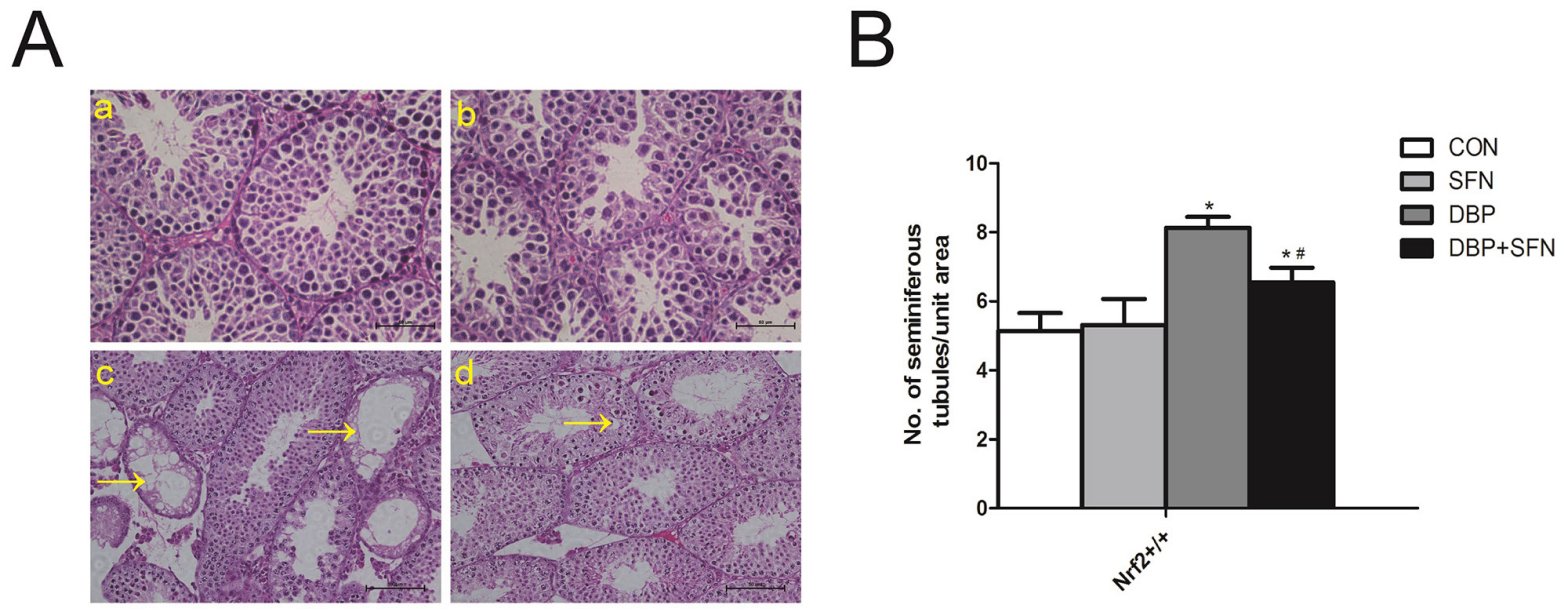
SFN prevents DBP-induced testicular oxidative stress injury in testicular morphology Testicular sections were stained with hematoxylin and eosin and examined using light microscopy at a magnification×400. **(A)** H&E staining of testis tissues in Nrf2^+/+^ mice. **(B)** Number of seminiferous tubule/unit area of testes in Nrf2^+/+^ mice. Data are expressed as mean ± SD. *significant difference vs. Control group (P < 0.05); #significant difference vs. DBP treated group (P < 0.05).

As shown in Figure 2A, DBP significantly attenuated testicular seminiferous epithelium injury and diminished the diameter of seminiferous tubules in Nrf2^+/+^ mice. Meanwhile, the cell layers of seminiferous tubules were scarce and irregularly arranged or even missing in DBP group. In partial vision, we found DBP led to the sparse arrangement of seminiferous tubules, Leydig cell hypoplasia and decreased number of Sertoli cells. Nevertheless, SFN supplementation preserved the tubular structure and arrangement of spermatogonial cells in Nrf2^+/+^ mice. Thus, SFN improved the histological injury induced by DBP. (Figure 2B).

### SFN decreases the number of DBP induced TUNEL positive cells

**Figure 3:**
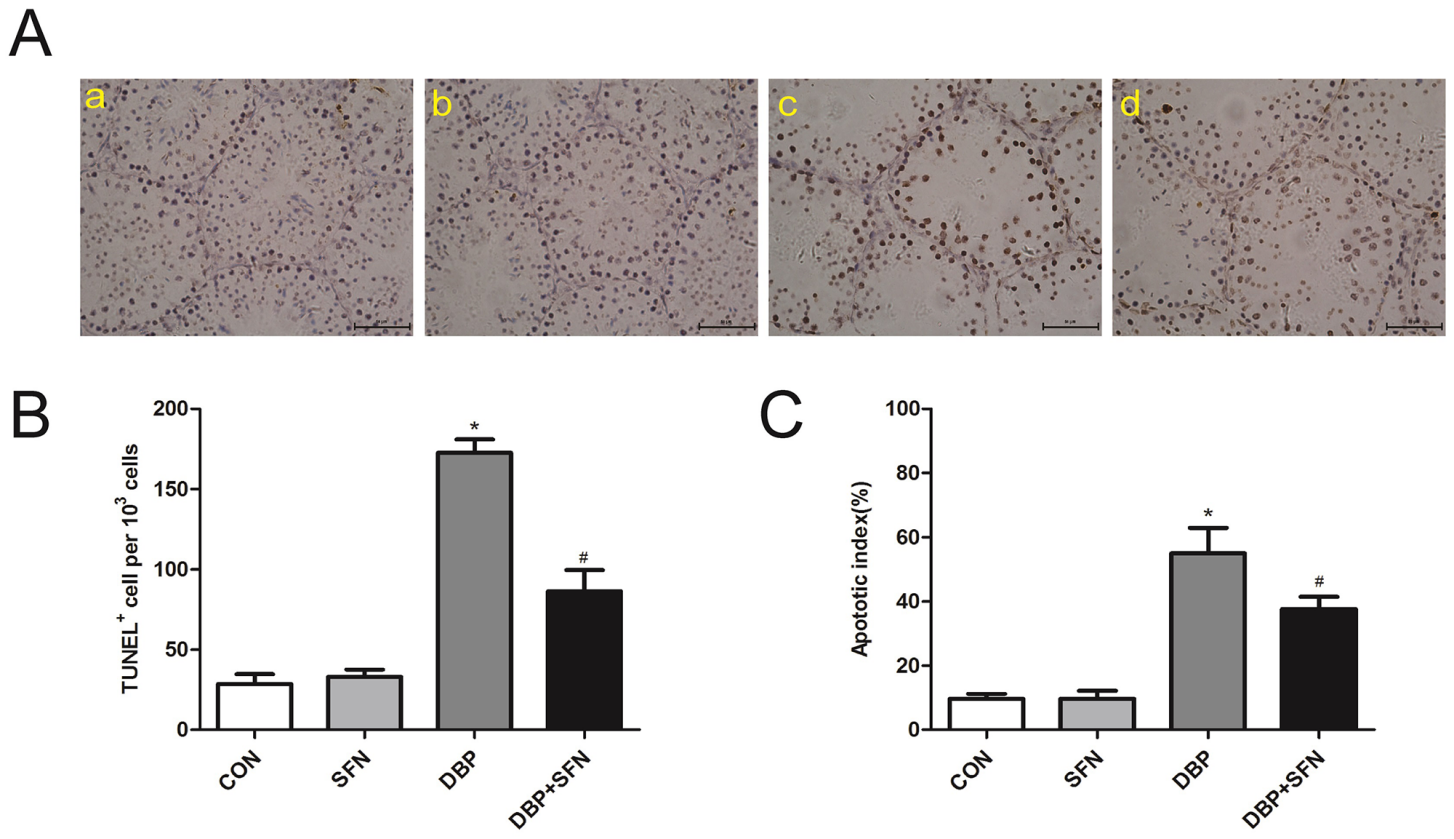
SFN decreases the number of TUNEL positive cells in response to DBP (×400) **(A)** TUNEL assay of testis tissues in Nrf2^+/+^ mice. Apoptotic cells exhibit a brown nuclear stain under microscope. **(B)** TUNEL positive cells per 10^3^ germ cells of testes in Nrf2^+/+^ mice. **(C)** Apoptotic index of testes in Nrf2^+/+^ mice. Data are shown as mean ± SD. *significant difference vs. Control group (P < 0.05); #significant difference vs. DBP treated group (P < 0.05).

TUNEL assay was performed to assess the extent to which SFN protected against apoptosis in the presence of DBP. Apoptosis was induced by DBP in the spermatogonia, primary and secondary spermatocytes. On the contrary, SFN protected against the DBP-induced apoptosis in Nrf2^+/+^ mice testes (Figure 3A). Semi-quantitative analysis by both total TUNEL positive cells/10^3^ germ cells and apoptotic index (AI) showed that DBP group had significantly higher incidence of testicular cell apoptosis than Control group (P<0.05). SFN treatment significantly decreased DBP-induced the number of TUNEL positive cells and AI in Nrf2^+/+^ mice (Figure 3B and 3C).

### SFN attenuates testicular oxidative stress injury

**Figure 4:**
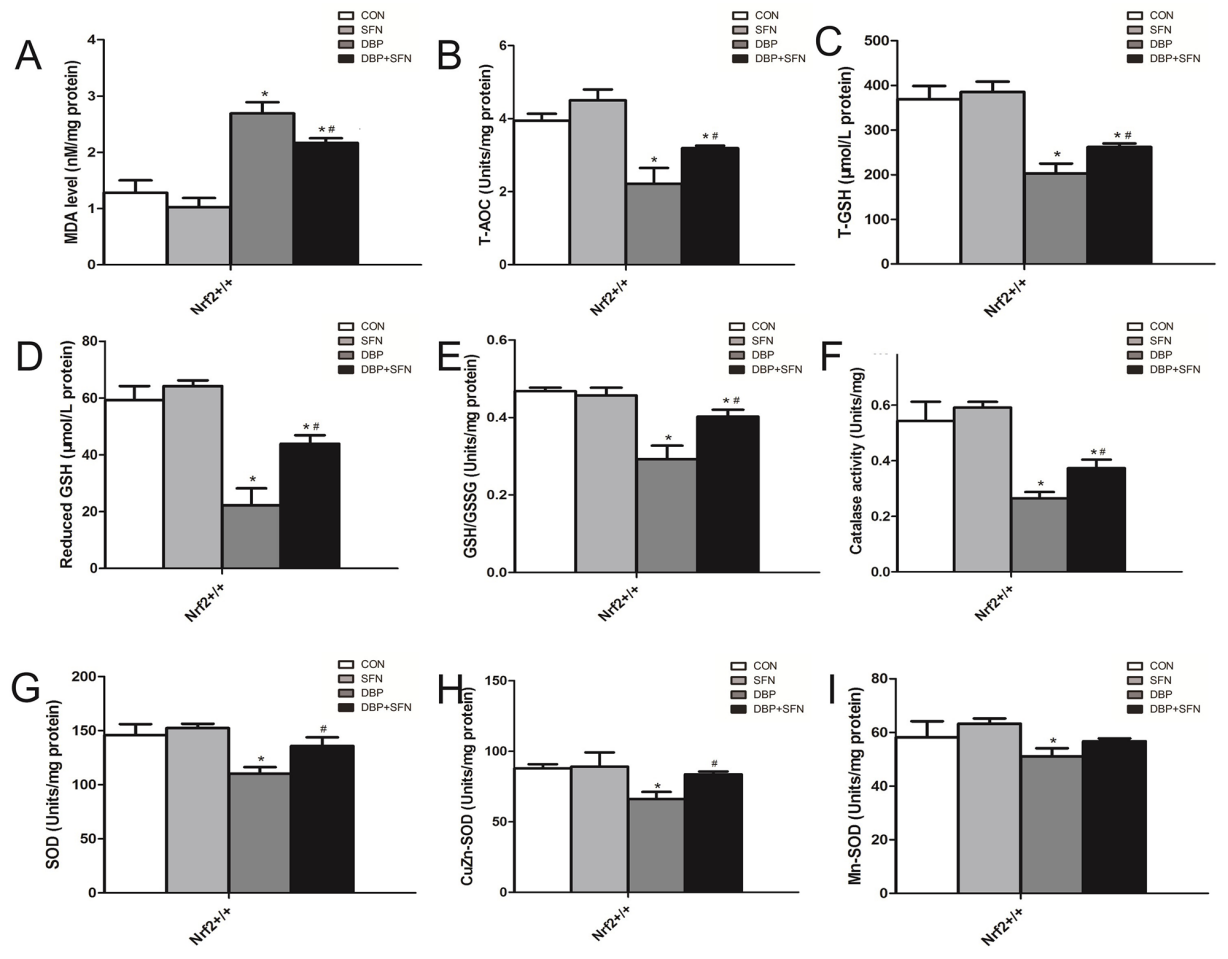
SFN attenuated oxidative stress injury by the assessment of biochemical parameters in Nrf2^+/+^ mice **(A)** Content of malondialdehyde (MDA) in testis tissues. **(B)** Content of total antioxidant capacity (T-AOC) in testis tissues. **(C-E)** Content of total glutathione (GSH), reduced GSH and GSH/GSSG in testis tissues. **(F)** Coentent of catalase activity in testis tissues. **(G-I)** Content of superoxide dismutase (SOD), CuZn-SOD and Mn-SOD in testis tissues. Data are expressed as mean±SD. *significant difference vs. Control group (P < 0.05); #significant difference vs. DBP treated group (P < 0.05).

As shown in Figure 4, compared with Control group, DBP group was significantly higher in Malondialdehyde (MDA) level. However, SFN supplementation could lower the level of MDA in Nrf2^+/+^ mice. Moreover, SFN could enhance the capacibility of antioxidant, such as total antioxidant capacity (T-AOC) and glutathione (GSH) and antioxidant enzymes, superoxide dismutase (SOD) and catalase (CAT) to reduce testicular oxidative stress injury, compared to DBP group in Nrf2^+/+^ mice. Therefore, these results indicated that Nrf2 played a protective role and its preactivation had a therapeutic effect on testicular oxidative stress injury.

### Effect of DBP and SFN supplementation on ROS generation

**Figure 5:**
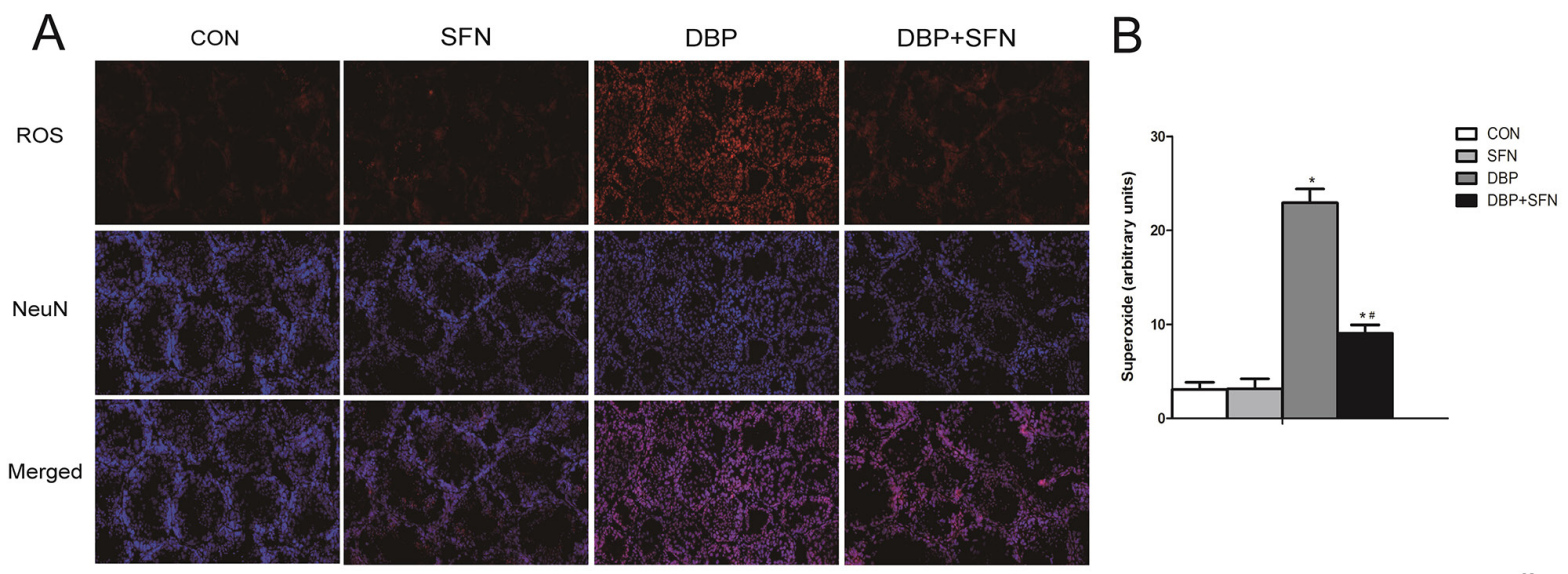
Effect of DBP stimulation and SFN supplementation on ROS generation **(A)** DHE staining of testis tissues in Nrf2^+/+^ mice. ROS exhibit red fluorescence under fluorescent microscope. **(B)** Density of ROS was reported as arbitrary units per millimetre square field. Data are expressed as mean ± SD. *significant difference vs. Control group (P < 0.05); #significant difference vs. DBP treated group (P < 0.05). Scale bar = 50 mm.

DHE reacts with superoxide radicals to form ethidium bromide, which in turn intercalates with DNA to provide nuclear fluorescence as a marker of ROS generation. As shown in Figure 5, DBP administration markedly increased the intensity of the DHE staining of testis, compared with Control and SFN group. However, the intensity of the fluorescent signal in the DBP + SFN group was significantly decreased compared with the DBP group in Nrf2^+/+^ mice. Thus, it suggested SFN protected against testicular oxidative stress injury induced by DBP via Nrf2.

### SFN supplementation influences the expression levels of Nrf2 and NF-κB in immunohistochemical staining

**Figure 6:**
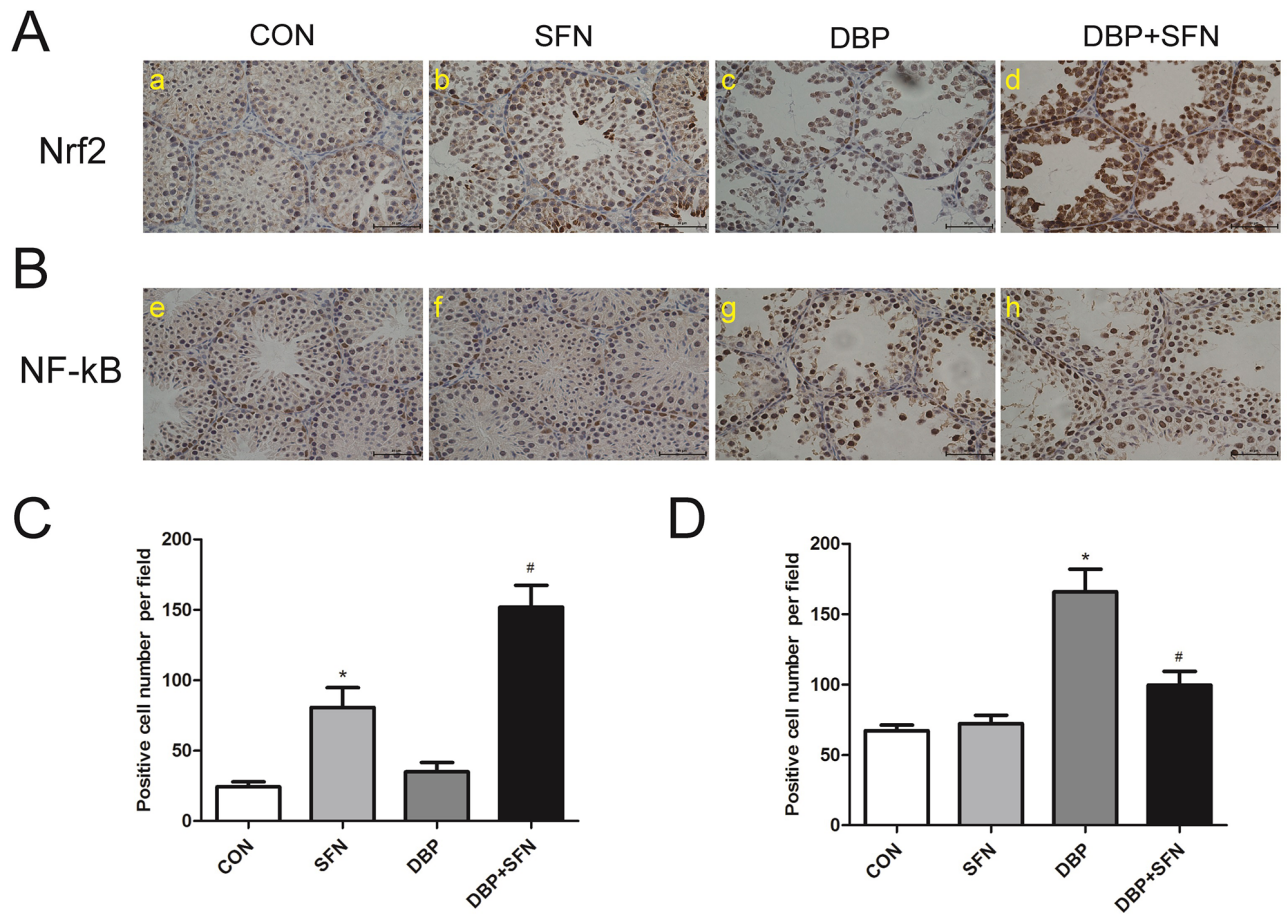
SFN supplementation influences expression levels of Nrf2 and NF-κB in immunohistochemical staining **(A)** Nrf2 expression levels in Nrf2^+/+^ mice. **(B)** statistical analyses of IHC results in Nrf2 expression levels in the different Nrf2^+/+^ groups. **(C)** NF-κB expression levels in Nrf2^+/+^ mice. **(D)** statistical analyses of IHC results in NF-κB expression levels in the different Nrf2^+/+^ groups. Data are expressed as mean ± SD. *significant difference vs. Control group (P < 0.05); #significant difference vs. DBP treated group (P < 0.05).

The expression levels of Nrf2 and NF-κB in the testes were examined by immunohistochemical analysis. High level of Nrf2 expression was detected in DBP + SFN groups, whereas there were low Nrf2 expression level in DBP treatment group in Nrf2^+/+^ mice (P<0.05) (Figure 6A and 6C). In addition, a significantly increased NF-κB expression was observed in DBP group compared with Control group (P<0.05), in Nrf2^+/+^ mice (Figure 6B and 6D). Collectively, these results suggested that SFN increased the expression level of Nrf2 to protect against testicular oxidative stress injury induced by DBP in male mice offsprings.

### SFN pretreatment increases expression levels of Nrf2 and its target genes protein expression

**Figure 7:**
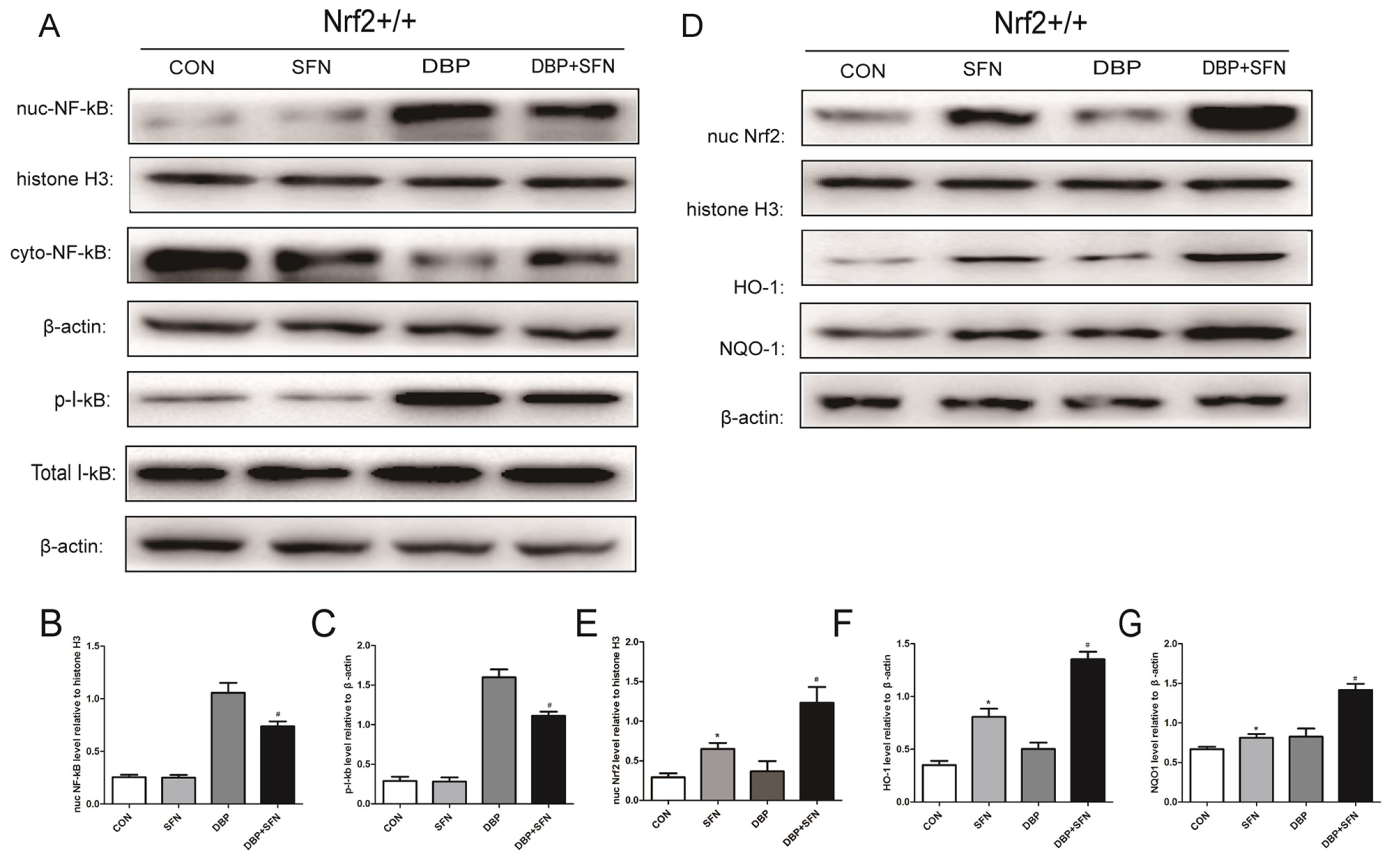
SFN pretreatment enhances Nrf2 nuclear translocation and increases HO-1 and NQO-1 protein expression in Nrf2^+/+^ mice **(A)** Protein expression levels of nuc NF-κB and p-I-kB in different groups. Histone H3 or β-actin was used as a protein Control to normalize volume of protein expression. **(B-C)** Protein levels were determined by densitometric analysis and normalized to the Histone H3 signal or the β-actin signal. **(D)** Protein expression levels of Nrf2, HO-1 and NQO-1 in different groups. Histone H3 or β-actin was used as a protein Control to normalize volume of protein expression. **(E-G)** Protein levels were determined by densitometric analysis and normalized to Histone H3 or the β-actin signal. Data are expressed as mean ± SD. *significant difference vs. Control group (P < 0.05); #significant difference vs. DBP treated group (P < 0.05).

Expression levels of Nrf2, NF-κB, I-kB, HO-1 and NQO-1 proteins were analyzed by western blotting. As shown in Figure 7, the expression level of Nrf2 and its downstream target genes HO-1 and NQO-1 in the testes of DBP+SFN group were dramatically increased compared with Control group and DBP group in Nrf2^+/+^ mice (P<0.05). In addition, the DBP administration in Nrf2^+/+^ mice obviously increased the expression level of NF-κB and the phosphorylation of I-kB, compared with Control group (P<0.05). However, SFN significantly decreased NF-κB and p-I-kB expression level in Nrf2^+/+^ mice. In consequence, the results indicated that Nrf2 might be associated with resisting oxidative stress and inflamation induced testicular damage.

### SFN pretreatment does not confer protection against DBP-induced testicular oxidative stress injury in the absence of Nrf2

**Figure 8:**
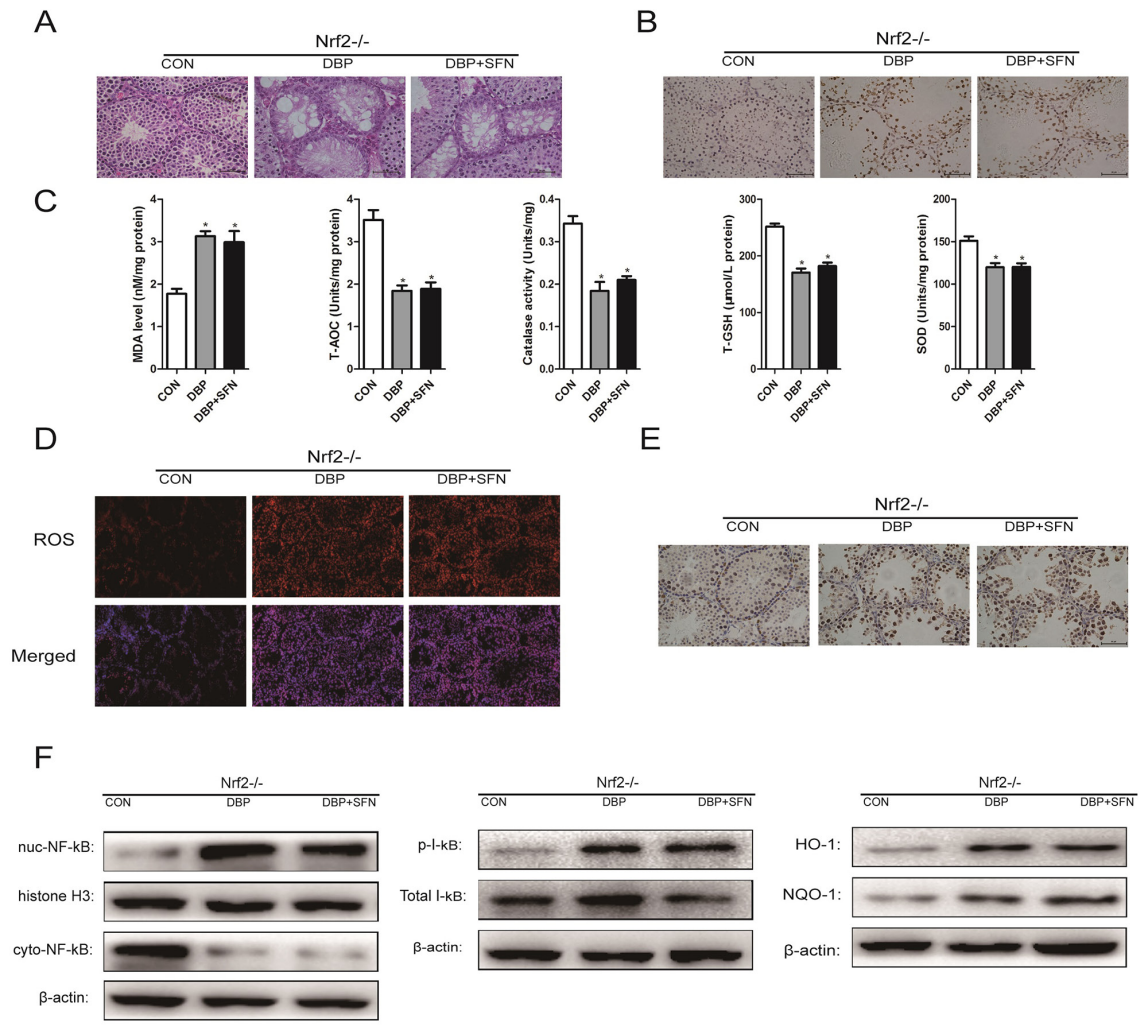
SFN pretreatment does not confer protection against DBP-induced testicular oxidative stress injury in Nrf2^-/-^ mice Nrf2^-/-^ mice were generated as previously described. Mice were administered either DBP+SFN or DBP or Control from GD 14 to GD19 in these female mice. **(A-F)** Mice receiving SFN showed no improvement in the tubular structure, DBP-induced apoptotic damage, the oxidative stress related parameters (MDA, T-AOC, CAT, T-GSH, SOD), ROS generation, inflammatory cytokine, NF-κB, I-kB and Nrf2 downstream target genes HO-1 and NQO-1 expression after receiving DBP when compared with mice receiving DBP treatment. Data presented are the mean fold change (FC) ± SD. from 5-10 mice per group.

To confirm that SFN acts primarily through the Nrf2 pathway, we examined the results of SFN treatment on mice deficient in Nrf2. Nrf2^-/-^ mice, age-matched littermates to Nrf2^+/+^ mice used in previous experiments (receiving DBP orally), were administered either DBP+SFN or DBP or Control from E14.5 to E19.5 in these female mice. Testes from SFN-treated and DBP-treated Nrf2^-/-^ offspring mice were harvested and were scored for tissue injury, as described previously. SFN did not confer improvement in the tubular structure and DBP-induced apoptosis in Nrf2 -/-mice (Figure 8A and 8B). Moreover, the oxidative stress related markers and ROS generation was not ameliorated by the administration of SFN in Nrf2^-/-^mice (Figure 8C and 8D). In addition, harvested testes were also analyzed by immunohistochemical staining and western blot for expression level of NF-κB and Nrf2 target genes. SFN did not decrease the production of pro-inflammatory cytokines NF-κB and the phosphorylation of I-kB, compared with mice receiving DBP, and it also failed to upregulate Nrf2 and its target genes in Nrf2^-/-^ mice, suggesting that SFN cytoprotection acts directly through Nrf2 (Figure 8E and 8F).

## DISCUSSION

EDCs, have been well described as anti-androgenic effects by interfering with the endocrine homeostasis, the action of endogenous hormones or other signaling components of the endocrine system during gestation, infancy and early childhood, thus increasing the risk of male reproductive dysfunction, such as hypospadias, cryptorchidism, and other birth defects [20]. As a derived form of phthalic acid, DBP proverbially existed in a variety of the household industries and consumer products, including plastics, paint, rubber, building materials, cosmetics and so on, thus resulting in ubiquitous daily exposures to humans [21-24]. Besides, DBP can easily leak out into the environment as it is not covalently bonded to the polymer matrix, bringing about its ubiquity in the environment [25]. Due to its special chemical structure, DBP can pass through the placental and blood-testis barriers. Previous study has shown that DBP could decrease fetal androgen production in utero exposure, which thereby leads to male reproductive dysfunction and an inhibition of masculinisation [34]. Therefore, DBP has drawn widespread media attention and regulatory scrutiny, which should be measured exposure in pregnant women, infants and children.

Because DBP can pass through placenta and it is essential for organ development in the embryonic period, maternal toxic accumulation can easily induce oxidative stress and tissue damage in multiple fetal organs. Thus, we suggest that DBP may cause testis injuries via oxidative stress. Importantly, we have previously carried out a series of experiments by stimulation of DBP on a cell model, and have found that DBP could cause testicular oxidative stress injury and antioxidant drugs might resist this damage [7]. Therefore, we used the pregnant mice as a research model to study the impact of exposure to DBP and explore the protective effects of SFN. Oxidative stress is one of the key mechanisms affecting the outcome through the generation of reactive oxygen species (ROS), such as the hydroxyl radical and singlet oxygen, which cause lipid peroxidation [26].

Oxidative stress is defined as a status in which the overproduction of ROS *in vivo* exceeds the removal capability of antioxidant enzymes and antioxidants, resulting in a local imbalance between ROS production and detoxification [27]. As one of the main indicator of oxidative stress, MDA is the end product of lipid oxidation [28]. In addition, T-AOC, GSH, SOD and CAT, play an important role in the efficient removal of excessive endogenous ROS [29]. In the present study, we found DBP induced testicular oxidative stress injury, which was confirmed by decreased capability of antioxidant defense system as well as increased MDA level, indicating that DBP induced male reproductive damage might be associated with oxidative stress. Meanwhile, the supplementation of SFN could ameliorate these damages and improve the testicular antioxidant status, contributing to a reasonable and important method for the resisting oxidative stress.

Nrf2, as a transcription factor encoded by the NFE2L2 gene, plays a crucial role in mediating the response of the endogenous antioxidant system, promoting the expression of antioxidant genes, inducing synthesis of phase II antioxidant and detoxification enzymes, which reacts with lipid peroixdation intermediates, hence protects against oxidative stress [30, 31]. However, the deficiency of Nrf2 increases the susceptibility of cells to oxidative stress damage [32]. Under normal or nonstressed conditions, the cytoplasmic Nrf2 is maintained at low level by Kelch-like ECH-associated protein 1 (Keap1) which degrade it through ubiquitin-mediated proteasomal degradation rapidly [33]. However, under oxidative stress conditions, Nrf2 is not degraded but translocates into the nucleus where it binds to ARE promoter, to trigger transcription of antioxidative genes and result in their protein levels elevation [34]. Moreover, NF-κB is a critical transcription factor that plays a significant role in apoptosis, inflammation and oxidative stress [35, 36]. Phosphorylated form of I-kB then activated NF-κB and enhancing its nuclear translocation in DBP-induced testicular oxidative stress injury. Along with the production of pro-inflammatory cytokines, DBP generates ROS. Nrf2 activation is closely associated with suppression of inflammation. Genetic ablation of Nrf2 in mice exacerbates DBP-induced testicular oxidative stress injury. Therefore, along with NF-κB, Nrf2 is being considered as a potential therapeutic target for the regulation of inflammatory diseases. In the present study, we not only reveal that Nrf2 expression increases by low dose of DBP stimulation but also demonstrate that SFN can significantly up-regulate Nrf2 expression and its downstream target genes, including HO-1 and NQO-1 to rescue testicular oxidative stress injury. Thus, Nrf2^−/−^ mice display increased NF-kB activation, the phosphorylation of I-kB and proinflammatory cytokine production [37, 38]. Furthermore, DBP further increased the NF-κB level and SFN significantly decreased its expression level.

As a potent activator of Nrf2, SFN is a naturally existing isothiocyanate derivative from its precursor glucoraphanin, which is first isolated and identified as a principal inducer of quinone reductase in broccoli and other Brassica species [39]. The biological impact of SFN, especially in constitutively regulating cytoprotective mechanisms in various tissues and cell types, underscores its potential health benefits [40, 41]. Once SFN is administered, Nrf2 can escape from its cytosolic repressor-- Keap1 mediated proteasomal degradation. Moreover, escaped Nrf2 translocates into nucleus to activate specific promoter sequences ARE containing genes, regulating the expression of a set of detoxifying and antioxidant enzymes [30, 42, 43]. In our research, we found that direct up-regulation of Nrf2 was capable of restoring the ROS stress, which supported that the use of SFN restored DBP induced testicular injury. Moreover, direct supplementation of SFN could only alleviate DBP-induced testicular oxidative stress injury in Nrf2^+/+^ mice, not in Nrf2^−/−^ mice.

## MATERIALS AND METHODS

### Ethics statement and animals

Nrf2^+/+^ mice (8-week-old C57BL/6J mice, weighting 20-24g) were procured from the Center for Experimental Animals at Nanjing Medical University (Nanjing, Jiangsu, China). In addition, Nrf2^−/−^ mice on C57BL/6J background were purchased by the Jackson laboratory (Bar Harbor, Maine, USA). Animals were maintained in our Experimental Animal Center with conventional housing conditions at room temperature (24 ± 2°C), with 50 ± 10% humidity and an automatically controlled 12-hours light/dark cycle under a pathogen-free condition. Besides, these mice were fed a standard rodent chow diet and drinking water during this experiment. They were acclimatized for one week before commencement of experiment. During the time of proestrus, virgin female mice were mated by 2:1 overnight with fertile male mice. Vaginal smears were examined each morning after the female mice were placed in cages with their male mates, and the morning of sperm detection was designated as embryonic day 0.5 (E0.5). Subsequently, genotype identification was carried out in these mice offsprings, which meant that the genotype of the mice offsprings was homozygous for Nrf2. The study was carried out in strict accordance with the recommendations in the National Institutes of Health Guide for the Care and Use of Laboratory Animals. All procedures conducted in experimental animals and the protocols were approved by the Committee on the Ethics of Animal Research in Animal Care Facility of Nanjing Medical University (approval number:14030145).

### Dose selection and animal model

Nrf2^+/+^ mice were respectively randomly divided into four groups each as follows: (1) Control group (n = 10), these timed-pregnant mice only were given corn oil (99.5% pure, Solvent Factory, Shanghai, China) by gavage from E14.5 to E19.5; (2) SFN group (n = 13), these female mice not only were given corn oil, but also 0.75 mg/kg·day SFN (Sigma-Aldrich, USA) by subcutaneous injection from E14.5 to E19.5; (3) DBP group (n = 7), these female mice were given DBP (Sigma-Aldrich, USA) by gastric intubation at a dose of 500 mg/kg body weight (bw)/day from E14.5 to E19.5; (4) DBP+SFN group (n = 11), these timed-pregnant mice were injected subcutaneously with 0.75 mg/kg·day SFN while simultaneously receiving DBP (same as DBP group). Body weight of each mouse was recorded daily. Subsequently, these mice offsprings were sacrificed to collect the testis samples in four weeks after birth. Briefly, the testes were dissected free of fat, washed with PBS and weighed after drying. One of samples was stored in 4% formaldehyde, and the other one was kept at −80 °C for subsequent biochemical measure. Besides, the AGD of each mouse was measured to calculate the ratio of AGD/body weight.

### Histological examination

Each harvested left testis tissue was immersed with 4% formaldehyde for 24 hours and dehydrated in ethanol and xylene, embedded in paraffin blocks, sectioned at 4-5 μm thickness. Then the chosen transverse sections from each sample were stained using hematoxylineosin (H&E) for quantification of various types of testicular cells. All histomorphological analyses described below were performed in blinded fashion. Then the sections were examined under light microscopy (Olympus BX-51, Tokyo, Japan) to evaluate testis structural changes and the number of seminiferous tubule/unit area of testes section by dividing the sum of number of tubule in each focus with total number of focus. Five focuses from each mouse were randomly examined.

### TdT-mediated dUTP nick-end labeling (TUNEL) assay

TUNEL assays were performed on 4% formaldehyde fixed testis sections by using In Situ Apoptosis Detection Kit (Roche, Basel, Switzerland) in accordance with the manufacturer’s instructions. The nucleus of any apoptotic cells would exhibit brown stains under a fluorescence microscope and were quantitatively counted manually in a blinded fashion. Results were presented as TUNEL positive cells per 10^3^ germ cells. The number of TUNEL-positive versus total cell nuclei were counted in five randomly chosen high-power fields (400×) containing 10^3^ cells on each slide to calculate an apoptotic index.

### Biochemical parameters

MDA, T-AOC, T-GSH, reduced GSH, GSH/GSSG, CAT, SOD, CuZn-SOD, Mn-SOD were estimated by using each Assay Kit (Jiancheng Bioengineering Institute, Nanjing, China), according to protocols described earlier [44].

### Detection of reactive oxygen species (ROS) level

ROS level assay was conducted as previously described [45]. For evaluating testicular intracellular superoxide production using In Situ Dihydroethidium (DHE, Sigma-Aldrich, USA) fluorescence, optimal cutting temperature media-embedded tissues were sectioned (5 μm) at -20 °C. After being fixed, tissues were incubated with 1 μm DHE in a light-protected, humidified chamber at room temperature for 30 min. The level of ROS by tissue was observed under a fluorescent microscope (Eclipse Ti-SR, Nikon Co., Japan). The density of the images was detected using a fluorescence spectrophotometer in arbitrary units per millimetre square field.

### Immunohistochemical staining

Testicular tissue sections were dewaxed, rehydrated, and washed 3 times for 5 minutes phosphate-buffered saline (PBS) (Gibco, Grand Island, USA). After slides were microwaved for 20 min and allowed to cool for 1 h at room temperature, endogenous peroxidase activity was blocked in all sections by incubating the sections in 3% H_2_O_2_ for 15 minutes. The sections were incubated with rabbit anti-mouse Nrf2 and nuclear factor kappa-light-chain-enhancer of activated B cells (NF-κB) antibody (Cell Signaling Technology, USA) overnight at 4 °C. Next day, the slides were washed and incubated with a horseradish peroxidase (HRP)-conjugated anti-goat secondary antibody (Cell Signaling Technology, USA) at a 1:100 dilution for 1 hour. After stained by DAB, the sections were observed under light microscopy.

### Western blot analysis

Cytoplasmic and nuclear proteins of freshly obtained testicular tissues were extracted using a Nuclear Extract Kit (Active Motif, Tokyo, Japan) according to the manufacturer’s instructions. Protein extracts separated upon 7.5% SDS-PAGE were transferred to 0.45 mm PVDF membrane (Bio-Rad, California, Hercules, USA). The membranes were incubated with TBST (20 mM Tris–HCL, pH 7.5, 150 mM NaCl, 0.1% Tween 20) with 5% nonfat milk powder for 2 hours before western blotting overnight at 4°C with rabbit polyclonal antibodies against mouse Nrf2, NF-κB, HO-1 and NQO-1 (Cell Signaling Technology, USA). Histone H3 and β-actin (Cell Signaling Technology, USA) were used as a protein Control to normalize volume of protein expression. After washed 3×10 minutes with TBST, the membranes were incubated with HRP-conjugated secondary antibody (1:2000, Cell Signaling Technology, USA) for 1.5 hours. Then after the membranes were washed 3 times with TBST, immunoreactive bands were visualized with electrochemiluminescence reagent (Amersham, Uppsala, Sweden). Densitometric and ImageQuant analysis were subsequently quantified analysis using Image Lab Software (Bio-Rad, USA).

### Statistical analysis

Statistical analyses were performed using analysis of variance (ANOVA), followed by the unpaired Student’s t test to evaluate the significance of differences between groups. All data are expressed as mean ± standard deviation (SD) for each group. The differences were evaluated using SPSS 22.0 (Armonk, New York, USA). Each experiment was performed at least 3 times with significant differences accepted when P values < 0.05.

## CONCLUSION

The present findings not only revealed that DBP induced male reproductive system in mice offsprings by excessive ROS, but also demonstrated that Nrf2 expression and its target genes were elevated in response to DBP stimulation. In addition, as a exogenous Nrf2 inducer, SNF could effectively protect against DBP-induced testicular oxidative stress injury through activation of Nrf2 signaling pathways in male mice offsprings. More further studies with different measuring methods will be warranted to recapitulate such results.
